# Revision of *Fothergilla* (Hamamelidaceae), including resurrection of *F. parvifolia* and a new species, *F. milleri*

**DOI:** 10.3897/phytokeys.144.49589

**Published:** 2020-03-17

**Authors:** Jake E. Haynes, Whitney D. Phillips, Alexander Krings, Nathan P. Lynch, Thomas G. Ranney

**Affiliations:** 1 Department of Plant and Microbial Biology, North Carolina State University, Raleigh, NC 27695, USA; 2 Department of Horticultural Science, North Carolina State University, Raleigh, NC 27695, USA; 3 Mountain Horticultural Crops Research and Extension Center, Mills River, NC 28759, USA

**Keywords:** Coastal Plain, Fothergilleae, Hamamelidoideae, southeastern United States

## Abstract

*Fothergilla* is a small genus of deciduous shrubs native to the southeastern United States that depending on circumscription comprises two to four species. Recent treatments recognized only two species in the genus: *F. gardenii* (tetraploid) and *F. major* (hexaploid). Until recently, no diploid taxon of *Fothergilla* was known. However, recent investigations identified a number of diploid populations in Alabama, Florida, Georgia, and South Carolina. A subsequent phylogenomic analysis showed that the diploids segregated into two, well-supported lineages, corresponding to largely allopatric populations. A re-examination of the morphology of diploid plants, in combination with the genetic evidence, has led us to the recognition of two species of diploids in the genus – a resurrected *F. parvifolia* and a new species (*F. milleri* W.D. Phillips & J.E. Haynes, **sp. nov.**) – bringing the total number of recognized species in *Fothergilla* to four. A revised taxonomic treatment of the genus is provided.

## Introduction

*Fothergilla* L. (Hamamelidaceae, Hamamelidoideae, Fothergilleae) is a small genus of deciduous shrubs native to the southeastern United States that depending on circumscription comprises two to four species (Small 1903; Britton 1905; Small 1913; Harms 1930; Small 1933; Bailey 1949; Weaver 1969). The genus was erected based on *F. gardenii* L., the latter based on material sent to Linnaeus by Dr Alexander Garden of Charleston, South Carolina (lectotype: LINN.-693.1). Loddiges and Cooke (1829) segregated *F. major* based on “having a spike of flowers, three inches or more in length; [...] later flowering, and [...] leaves [...] very broad, and much more toothed” (Weaver 1969). Subsequently, two more names were validly published at the rank of species: *F. monticola*Ashe (1897) and *F. parvifolia* Kearney in Small (1903), a segregate of *F. gardenii*.

Ashe (1897) published the name *Fothergilla monticola*, under the mistaken impression that *F. major* Lodd. represented a robust form of coastal *F. gardenii*, rather than applied to the primarily mountain populations of *Fothergilla*. This circumscription was followed by Hesse (1909), Rehder (1910), Bailey (1949), Harms (1930), Anderson and Sax (1935), and Ernst (1963), but not Small (1903, 1913, 1933) or Radford et al. (1968), who recognized only *F. major*. In revising the genus, Weaver (1969) recognized *F. monticola* as a synonym of *F. major*, a treatment that has been followed ever since (Meyer 1997; Weakley 2015).

Kearney (in Small 1903) segregated *Fothergilla parvifolia* from *F. gardenii* on the basis of leaf width (about as broad as long in *F. parvifolia* vs. longer than broad in *F. gardenii*), leaf base (cordate in *F. parvifolia* vs. cuneate to rounded in *F. gardenii*), and leaf margin (toothed from below the middle to the apex in *F. parvifolia* vs. toothed only near the apex in *F. gardenii*). This circumscription was followed by Britton (1905), Small (1913, 1933), and Harms (1930), though more recent authors apparently found these characters uninformative and treated *F. parvifolia* as a synonym of *F. gardenii* (Weaver 1969; Meyer 1997; Weakley 2015). In fact, recent treatments recognized only two species in the genus: *F. gardenii* (incl. *F. parvifolia*) and *F. major* (incl. *F. monticola*) (Weaver 1969; Meyer 1997; Weakley 2015).

As circumscribed by most recent authors, *Fothergilla gardenii* is found in wet savannas and pocosins in the coastal plains of North Carolina, South Carolina, Georgia, Florida, and Alabama, whereas *F. major* occurs primarily in woodlands, bluffs, and riverbanks of the upper Piedmont and mountains of North Carolina, South Carolina, Georgia, Alabama, Tennessee, and Arkansas (Weaver 1969; Meyer 1997; Weakley 2015). Recent authors generally distinguish *F. gardenii* from *F. major* by the smaller stature (3–10 dm vs. 10–80 dm in *F. major*), smaller leaves (<5.2 cm wide vs. > 5.2 cm wide in *F. major*), leaf dentations (tending towards the upper half of the leaf vs. extending below the middle in *F. major*), base symmetry (symmetric vs. asymmetric in *F. major*), hypanthium length (3–4.5 mm vs. 4–9.2 mm in *F. major*), number of stamens per flower (12–24 vs. 22–32 in *F. major*), and seed size (4.8–6.3 mm long vs. 6.2–7.8 mm long in *F. major*) (Radford et al. 1968; Weaver 1969; Weakley 2015). Weaver (1969) recognized *Fothergilla gardenii* as a tetraploid with 2*n* = 4*x* = 48, and *F. major* as a hexaploid with 2*n* = 6*x* = 72.

Although *F. major* and *F. gardenii* sensu Weaver (1969) have allopatric distributions, they have been grown together in cultivation, where they will freely hybridize. Ranney et al. (2007) concluded that the majority of cultivars represented in commerce was pentaploid with (2*n* = 5*x* = 60) and named the nothospecies *F. × intermedia* Ranney & Fantz, a finding that cleared up previous controversy as to whether common cultivars (such as ‘Mount Airy’) represented *F. major* or *F. gardenii*. No pentaploids have been identified in nature.

Until recently, no diploid taxon of *Fothergilla* was known. However, recent sampling and cytometric analysis identified a number of diploid populations in Alabama, Florida, Georgia, and South Carolina (Ranney et al. 2012). This work was followed by phylogenomic analyses, examining the origins of *F. gardenii* and *F. major* and their relationship to the diploid populations (Qi et al. 2015). These analyses identified 11 haplotypes of plastid DNA, five of ETS, and 13 of combined plastid-ETS sequences. Of these, no haplotypes were shared between the diploid populations and polyploid taxa (i.e., *F. gardenii* and *F. major*). Furthermore, the diploid OTUs segregated into two, well-supported lineages, corresponding to largely allopatric populations. A re-examination of the morphology of diploid plants, in combination with the genetic evidence, has led us here to the recognition of two species of diploids in the genus: a resurrected *F. parvifolia* and a new species (*F. milleri*) as described below. A revised taxonomic treatment of the genus is provided.

## Methods

**Figure 1.**
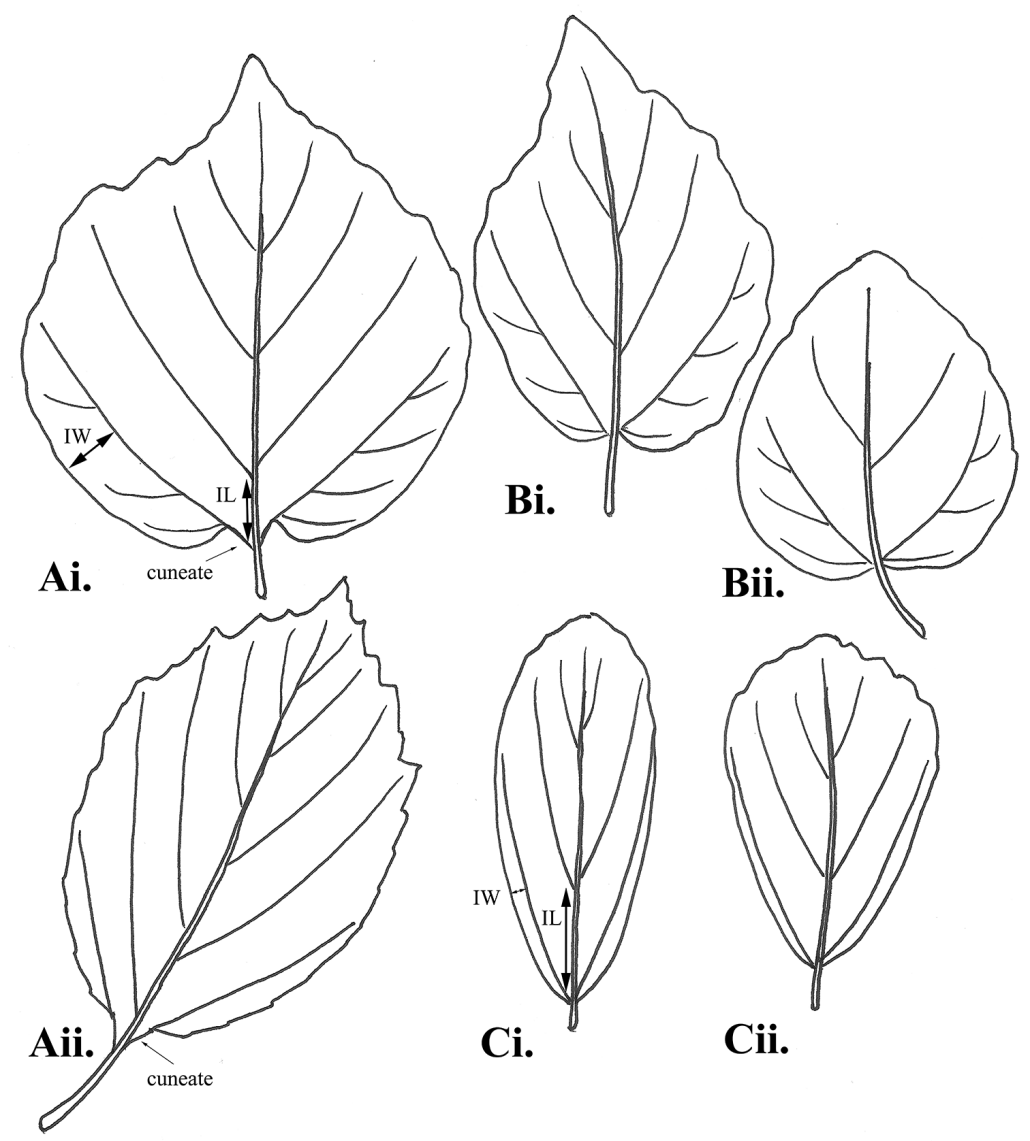
Leaf shape and base variation in *Fothergilla*: **Ai–Aii***Fothergilla major* (V-cordate/V-rounded, i.e. with distinct cuneate portion at extreme blade base that broadens out laterally to cordate [Ai.] or rounded [Aii.]) **Bi–Bii***Fothergilla parvifolia* (note the neatly cordate bases without well-defined cuneate portions at extreme blade base) **Ci–Cii***Fothergilla milleri* (bases rounded). *Fothergilla gardenii* not shown as bases are variably rounded, cuneate, or cordate. IL = the length of the midvein interval between the junction of the midvein and lowermost secondary vein and the junction of the midvein and the next-most distal secondary vein on the same side of the leaf; IW = the width, at the widest point, of the intervening leaf surface between the lowermost secondary vein and the leaf margin. Illustrations by A. Krings based on *Lynch 29* (Ai.), *Lynch 21* (Aii.), *Phillips 56* (Bi.), *Lynch 18* (Bii.), *Lynch 68* (Ci.), and *Lynch 69* (Cii.).

Specimens studied in the course of preparing this revision included: (1) 34 accessions of *Fothergilla* from throughout the southeastern United States, planted and grown in a common garden at the Mountain Horticultural Crops Research and Extension Center in Mills River, North Carolina (Table 1), and (2) 207 specimens from the following herbaria: AUA, BRIT/VDB, DOV, F, FLAS, GEO, HTTU, KNK, LINN, MISS, MO, NCSC, NCU, NY, OS, US, and UWFP. The accessions from the Mountain Horticultural Crops Research and Extension Center are particularly important because ploidy level is known for all individuals (Table 1). The Phylogenetic Species Concept (PSC) sensu Nixon and Wheeler (1990), and its method of discovery–Population Aggregation Analysis–was applied to determine if taxa could be recognized. Thirteen binary and three multi-state morphological characters were assessed (Table 2). These included several novel characters not heretofore explicitly employed in study of the genus, such as the orientation of blades on living plants (e.g., spreading, erect, or drooping) and the ratio of: (1) the width, at the widest point, of the intervening leaf surface between the lowermost secondary vein and the leaf margin (IW), and (2) the length of the midvein interval between the junction of the midvein and lowermost secondary vein and the junction of the midvein and the next-most distal secondary vein on the same side of the leaf (IL; Fig. 1). To determine the IW:IL ratio for the Population Aggregation Analysis, respective measurements were taken from the largest measurable leaf of each of 34 accessions of known ploidy (Table 1). Subsequent to our post-analysis decisions regarding taxon recognition, we took additional IW and IL measurements from 96 loaned herbarium specimens (marked by ^m^ in the list of exsiccatae below). We also searched the SERNEC portal (http://sernecportal.org) to identify any additional specimens of the diploid taxa we recognized. This search resulted in six additional specimens, which we added to the list of exsiccatae in the taxonomic treatment below (only two of these represented a county not represented in our original loan of specimens). The combined sets of specimens of known ploidy (Table 1) and available to us from the herbaria identified above were the source of the morphological data we provide in our species descriptions.

**Table 1.** Accessions grown at the Mountain Horticultural Crops Research and Extension Center and examined in the present study. F# and genome size as reported from Qi et al. (2015).

F#	Accession	Ploidy	Species sensu Weaver (1969)/Meyer (1997)	Species sensu present study	Native Location	Genomic Size (pg) +/- Standard Error
01	2011-083	2x	*F. gardenii*	*F. milleri*	Okaloosa Co., FL	1.70 +/- 0.03
02	2011-087	2x	*F. gardenii*	*F. milleri*	Baldwin Co., AL	1.78 +/- 0.02
03	2011-088	2x	*F. gardenii*	*F. milleri*	Walton Co., FL	1.74 +/- 0.00
04	2011-168	2x	*F. gardenii*	*F. parvifolia*	Tattnall Co., GA	1.74 +/- 0.05
05	2011-170	2x	*F. gardenii*	*F. parvifolia*	Emanuel Co., GA	1.75 +/- 0.10
06	2011-171	2x	*F. gardenii*	*F. parvifolia*	Long Co., GA	1.73 +/- 0.02
07	2011-178	2x	*F. gardenii*	*F. milleri*	Taylor Co., GA	1.74 +/- 0.02
08	2012-060	2x	*F. gardenii*	*F. milleri*	Walton Co., FL	1.76 +/- 0.01
09	2012-084	2x	*F. gardenii*	*F. parvifolia*	Aiken Co., SC*	1.82 +/- 0.04
11	2011-085	4x	*F. gardenii*	*F. gardenii*	Richmond Co., NC	3.69 +/- 0.02
12	2011-096	4x	*F. gardenii*	*F. gardenii*	Carteret Co., NC	3.64 +/- 0.08
13	2011-097	4x	*F. gardenii*	*F. gardenii*	Hoke Co., NC	3.57 +/- 0.00
14	2011-103	4x	*F. gardenii*	*F. gardenii*	Carteret Co., NC	3.69 +/- 0.00
15	2011-123	4x	*F. gardenii*	*F. gardenii*	Richmond Co., NC	3.68 +/- 0.04
16	2012-075	4x	*F. gardenii*	*F. gardenii*	Charleston Co., SC	3.40 +/- 0.01
17	2012-076	4x	*F. gardenii*	*F. gardenii*	Horry Co., SC	3.33 +/- 0.16
18	2012-077	4x	*F. gardenii*	*F. gardenii*	Charleston Co., SC	3.76 +/- 0.05
19	2012-078	4x	*F. gardenii*	*F. gardenii*	Effingham Co., GA	3.61 +/- 0.02
20	2008-009	6x	*F. major*	*F. major*	Dekalb Co., AL	5.27 +/- 0.02
21	2011-082	6x	*F. major*	*F. major*	Searcy Co., AR	5.22 +/- 0.12
22	2011-091	6x	*F. major*	*F. major*	Oconee Co., SC	5.40 +/- 0.04
23	2011-092	6x	*F. major*	*F. major*	Marshall Co., AL	5.23 +/- 0.11
24	2011-093	6x	*F. major*	*F. major*	Blount Co., AL	5.29 +/- 0.03
25	2011-105	6x	*F. major*	*F. major*	Burke Co., NC	5.09 +/- 0.05
26	2011-112	6x	*F. major*	*F. major*	Transylvania Co., NC	5.12 +/- 0.02
28	2011-122	6x	*F. major*	*F. major*	Montgomery Co., NC	5.27 +/- 0.06
29	2011-124	6x	*F. major*	*F. major*	Orange Co., NC	5.15 +/- 0.10
30	2011-131	6x	*F. major*	*F. major*	Transylvania Co., NC	5.13 +/- 0.05
31	2011-146	6x	*F. major*	*F. major*	Walker Co., GA	5.36 +/- 0.02
32	2011-147	6x	*F. major*	*F. major*	Marshall Co., AL	5.17 +/- 0.05
33	2011-163	6x	*F. major*	*F. major*	Rutherford Co., NC	5.27 +/- 0.01
34	2011-164	6x	*F. major*	*F. major*	Lumpkin Co., GA	5.31 +/- 0.01
35	2011-169	6x	*F. major*	*F. major*	Fulton Co., GA	5.17 +/- 0.17
36	2012-065	6x	*F. major*	*F. major*	Scott Co., TN	5.24 +/- 0.28

* Reportedly from Aiken County, SC, but the population could not be relocated in a recent survey.

**Table 2.** Binary and multi-state morphological character states assessed in *Fothergilla*.

Character	Character State
1. Stem: Pubescence color	(0) White/Gray, (1) Brown
2. Leaf color	(0) Green, (1) Blue-green/Gray-green
3. Leaf orientation	(0) Spreading, neither drooping nor erect, (1) Erect, (2) Drooping
4. Leaf shape	(0) Obovate, (1) Ovate
5. IW:IL ratio	(0) < 0.5, (1) > 0.5
6. Leaf base	(0) Cordate, (1) V-cordate/V-rounded, (2) Rounded/truncate, (3) Cuneate
7. Leaf apex	(0) Acute, (1) Obtuse
8. Leaf dentation	(0) Top 1/3 of leaf only, (1) Begins at middle, (2) Begins below middle
9. Leaf dentation type	(0) Crenate, (1) Dentate/Serrate
10. Lamina: Adaxial waxy bloom	(0) Present (i.e., lamina glaucous), (1) Absent
11. Lamina surface: Pubescence color	(0) White/Gray, (1) Brown
12. Lamina midvein: Pubescence color	(0) White/Gray, (1) Brown
13. Floral bract color	(0) Tan/White/Pink, (1) Dark Brown
14. Constriction between filament and anther	(0) Present, (1) Absent
15. Style pubescence	(0) Present, (1) Absent
16. Seed apex shape	(0) Rounded/Obtuse, (1) Acute/Acuminate

## Results and discussion

**Figure 2.**
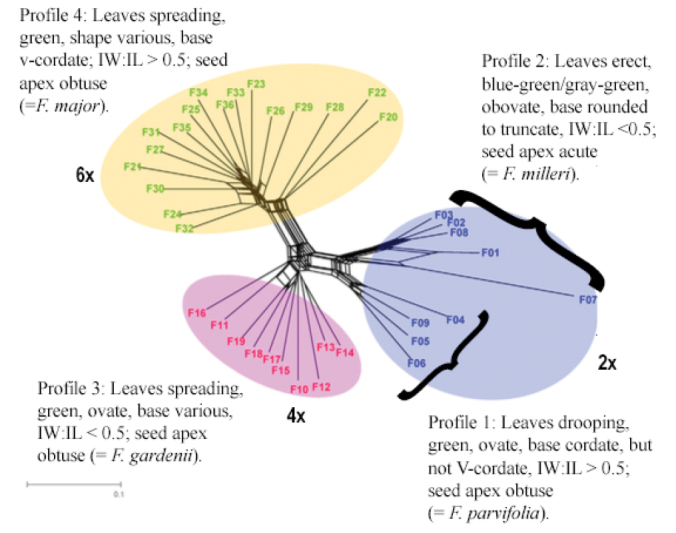
Phylogenetic network of *Fothergilla* cytotypes from Qi et al. (2015; generated by NeighborNet method using SplitTree 4 based on 165 SNP dataset), corresponding morphological population aggregation profiles, and associated names recognized herein (Table 3). For accession details, including localities, see Table 1.

**Figure 3.**
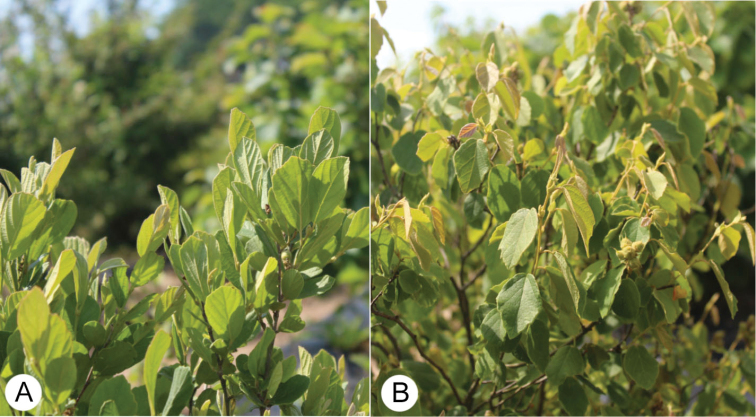
The two leaf orientations found in the diploid lineages of *Fothergilla***A** erect leaves of *F. milleri* (aggregate profile 2) **B** drooping leaves of *F. parvifolia* (aggregate profile 1). Photos by J. Haynes of plants at the Mountain Horticultural Crops Research and Extension Center, Mills River.

Population Aggregation Analysis revealed four distinct aggregate profiles, each corresponding to one of the major lineages identified by Qi et al. (2015) (Table 3; Fig. 2). In contrast to the tetraploids and hexaploids, which bear leaves mostly spreading (profiles 3 and 4), diploids bear leaves either distinctly drooping (profile 1) or erect (profile 2) (Fig. 3). Diploids with drooping leaves (profile 1) also exhibit green, ovate laminas with marginal dentation beginning at the middle of the blade, as well as obtuse seed apices, in contrast to diploids bearing erect leaves (profile 2), which exhibit blue-green or gray-green, obovate laminas with marginal dentation beginning only at the top third of the blade and seed apices that are acute.

**Figure 4.**
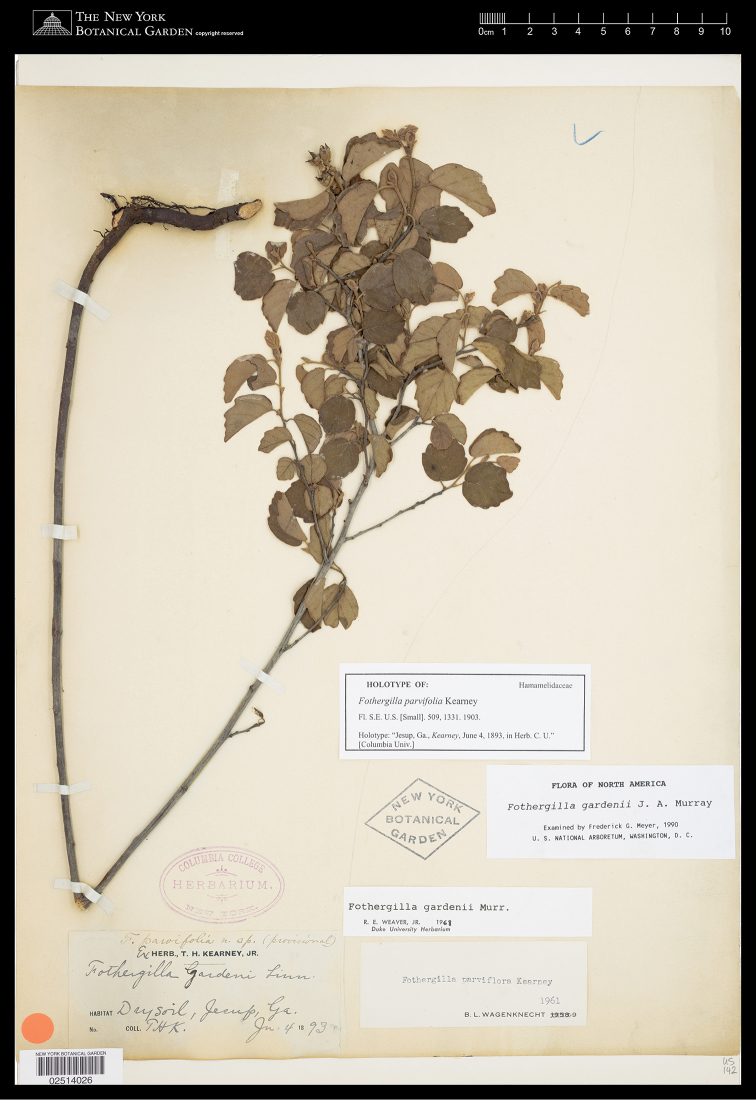
Holotype of *Fothergilla parvifolia* Kearney (*Kearney s.n.*, NY-02514026). Note the identifying characteristics of *F. parvifolia*: leaves drooping, bases neatly cordate, and margins with the crenation/serration from the midpoint to the apex.

A re-examination of type material revealed that a name already exists for the drooping-leaved diploids representing profile 1: *F. parvifolia* Kearney (holotype: *Kearney s.n.*, NY-02514026; Fig. 4). This name was originally published in Small (1903), but lumped beneath *F. gardenii* by subsequent authors (Ernst 1963; Radford et al. 1968; Weaver 1969; Meyer 1997 and Weakley 2015). The mean IW:IL ratio for *F. parvifolia* accessions from the common garden at the Mountain Horticultural Crops Research and Extension Center (Table 1; known ploidy) is 0.86 (s.d. = 0.25, n = 4) and 0.96 (s.d. = 0.22, n = 7) when including additional herbarium specimens. There are no prior names applicable to plants referred to profile 2, which we here recognize as representing a new species, described as *F. milleri* below. The mean IW:IL ratio for *F. milleri* accessions from the common garden at the Mountain Horticultural Crops Research and Extension Center (Table 1; known ploidy) is 0.26 (s.d. = 0.13, n = 4) and 0.29 (s.d. = 0.10, n = 14) when including additional herbarium specimens.

*Fothergilla gardenii* is the appropriate name for the tetraploids representing profile 3. With the removal of the diploid components previously lumped under that name, our taxon concept of *F. gardenii* is necessarily narrower than that of recent authors such as Weaver (1969) and Meyer (1997). The mean IW:IL ratio for *F. gardenii* accessions from the common garden at the Mountain Horticultural Crops Research and Extension Center (Table 1; known ploidy) is 0.29 (s.d. = 0.09, n = 10) and 0.33 (s.d. = 0.09, n = 49) when including additional herbarium specimens.

*Fothergilla major* is the appropriate name for the hexaploids referred to profile 4. The average IW:IL ratio for *F. major* accessions from the common garden at the Mountain Horticultural Crops Research and Extension Center (Table 1; known ploidy) is 0.99 (s.d. = 0.37, n = 16) and 0.96 (s.d. = 0.32, n = 60) when including additional herbarium specimens. Our taxon concept of *F. major* is consistent with that of Weaver (1969) and Meyer (1997). However, it should be noted that although the leaf bases of *F. major* have often been generally described as cordate or rounded (if sometimes asymmetrically so), the shape is actually a combination of a short cuneate section adjoining the petiole that broadens out laterally into the more general cordate or rounded shape. We here use the terms V-cordate and V-rounded to describe this type of base (the “V” representing the cuneate section) and consider it structurally distinct from the neatly cordate leaf bases of *F. parvifolia* (Fig. 1).

Based on these results, *Fothergilla* is here recognized as a genus of four species endemic to the southeastern United States: *F. gardenii* (4x), *F. major* (6x), *F. milleri* (2x), and *F. parvifolia* (2x). An updated taxonomic treatment follows.

**Table 3.** Population Aggregation Analysis population profiles for *Fothergilla* and corresponding taxa. (–) = populations exhibited mixture of characters.

Attributes								
	Seed apex	Leaf color	Leaf orientation	Leaf shape	Leaf base	Leaf dentation	IW:IL ratio	Ploidy
Aggregate profile 1 (*F. parvifolia*)	Obtuse	Green	Drooping	Ovate	Cordate (but not V-cordate)	Begins at middle	> 0.5	2x
Aggregate profile 2 (*F. milleri*)	Acute	Blue-green/gray-green	Erect	Obovate	Rounded to truncate	Only top 3^rd^ of leaf	< 0.5	2x
Aggregate profile 3 (*F. gardenii*)	Obtuse	Green	Spreading	Ovate	–	–	< 0.5	4x
Aggregate profile 4 (*F. major*)	–	Green	Spreading	–	V-cordate/V- rounded	Begins below middle	> 0.5	6x

### Taxonomic treatment

#### Fothergilla

Taxon classificationPlantaeSaxifragalesHamamelidaceae

L., Syst. Veg. ed. 13. 418. 1774

F9AB60B8-9179-5DF2-813D-AEFB92A8716D

##### Type.

*Fothergilla gardenii* L., Syst. Veg. ed. 13. 418. 1774 [as *F. Gardeni*] (***Lectotype***: LINN-693.1, LINN [online!], designated by Reveal in Jarvis et al. 1993).

##### Description.

***Shrubs***, rhizomatous, perennial, to 8 m tall; clump forming, usually multi-stemmed. ***Bark*** smooth, gray to reddish-brown. ***Stems*** stellate-pubescent when young, sparsely pubescent to glabrate when mature. ***Vegetative buds*** naked, densely stellate-pubescent. ***Leaves*** deciduous, simple, alternate, petiolate; stipules lanceolate to ovate; blades pinnately-veined, ovate, obovate, or oblong, bases oblique to symmetric, rounded, truncate, V-cordate or cordate, margins crenate to serrate from at or below middle to apex, or only near apex, apices acute to obtuse, surfaces sparsely to densely stellate-pubescent or glabrous, abaxially glaucous or not. ***Inflorescence*** terminal, spikes, erect, appearing with or before leaves. ***Flowers***: mostly perfect, proximal often staminate; calyx lobes 5–7, connate, forming shallow hypanthium; apetalous; stamens 10–32, adnate to hypanthium, filaments white, anthers yellow, basifixed, 2-loculed; gynoecium, adnate to hypanthium, 2-carpellate, connate below, divergent near apex into separate styles, semi-inferior. ***Fruit***: capsules, in groups of 3 or more, loculicidal, gray to brown, densely stellate-pubescent throughout with long, simple trichomes mixed in predominantly on and above persistent hypanthium; remnant style beaks conspicuous, abscising with maturity. ***Seeds***: 2 per capsule, glossy, hard, nearly white, mottled, or solid red-brown to brown, ellipsoid to slightly ovoid, round to slightly flattened near apex in cross-section, apex round, obtuse, or acute to acuminate, when acuminate often recurved.

##### Notes.

*Fothergilla* seeds vary in color and shape, but the surface texture is consistent throughout. The seed surface is smooth and glossy with a conspicuous hilum scar at the base. In *F. major*, seeds are variable in color: completely white, mottled, or brown. In *F. gardenii*, seeds are mottled white-brown, with white conspicuously appearing around the margins of the seeds. In both diploid taxa, seeds are consistently red-brown to brown. In *F. gardenii* and *F. major*, it appears that color is affected by age and storage, as older herbaria specimens have nearly completely white seeds.

### Key to the species of *Fothergilla*

**Table:** 

1	Leaves spreading	**2**
–	Leaves erect or drooping	**3**
2	Shrub usually <1 m tall; leaf blades narrowly ovate to ovate, to 5.3 cm wide, bases usually rounded to cuneate, sometimes shallowly cordate, IW:IL ratio < 0.5; petioles 3.9–10.5 mm long; Coastal Plain (Carolinas and e Georgia)	**1. *F. gardenii***
–	Shrub usually >1 m tall and often taller (to 8 m); leaf blades broadly ovate or elliptic to suborbiculate, to 13.0 cm wide, bases usually V-cordate or V-rounded, IW:IL ratio > 0.5; petioles 8.0–17.1(–18.9) mm long; mountains and Piedmont (rarely in Coastal Plain-like seep communities)	**2. *F. major***
3	Leaves erect, blue-green or gray-green, blades obovate, bases rounded to truncate, margins crenate to serrate above middle, mainly near apex, IW:IL ratio < 0.5, petioles 1/3–1/2 the length of the IL; seed apex acute to acuminate, if acuminate often recurved; Alabama, nw Florida, and w Georgia	**3. *F. milleri***
–	Leaves drooping, mostly green, blades ovate, bases cordate, margins coarsely crenate to serrate mostly from the middle to the apex, IW:IL ratio > 0.5, petioles nearly as long to longer than the IL; seed apex obtuse; Georgia and South Carolina	**4. *F. parvifolia***

### Alternate key to the species of *Fothergilla*

**Table:** 

1	IW:IL ratio < 0.5	**2**
–	IW:IL ratio > 0.5	**3**
2	Leaves spreading, blades green, narrowly ovate to ovate, petioles usually ¾ the length of the IL or longer; seed apex rounded or obtuse; Carolinas and e Georgia	**1. *F. gardenii***
–	Leaves erect, blades blue-green or gray-green, obovate, petioles 1/3–1/2 the length of the IL; seed apex acute to acuminate, if acuminate often recurved; Alabama, nw Florida, and w Georgia	**3. *F. milleri***
3	Leaves spreading, bases usually V-cordate or V-rounded; Piedmont and Mountains (rarely in Coastal Plain-like seep communities)	**2. *F. major***
–	Leaves drooping, bases cordate; Coastal Plain (Alabama, Georgia, South Carolina)	**4. *F. parvifolia***


#### 1. Fothergilla gardenii

Taxon classificationPlantaeSaxifragalesHamamelidaceae

L. Syst. Veg. ed. 13. 418. 1774 [as F. gardeni]

B7BBD1B7-5459-5337-98EA-EDC0439CBE3B

Hamamelis virginiana L. var. carolina L., Mant. 2: 333. 1771. Type. Unknown.

##### Type.

**Figure 5.**
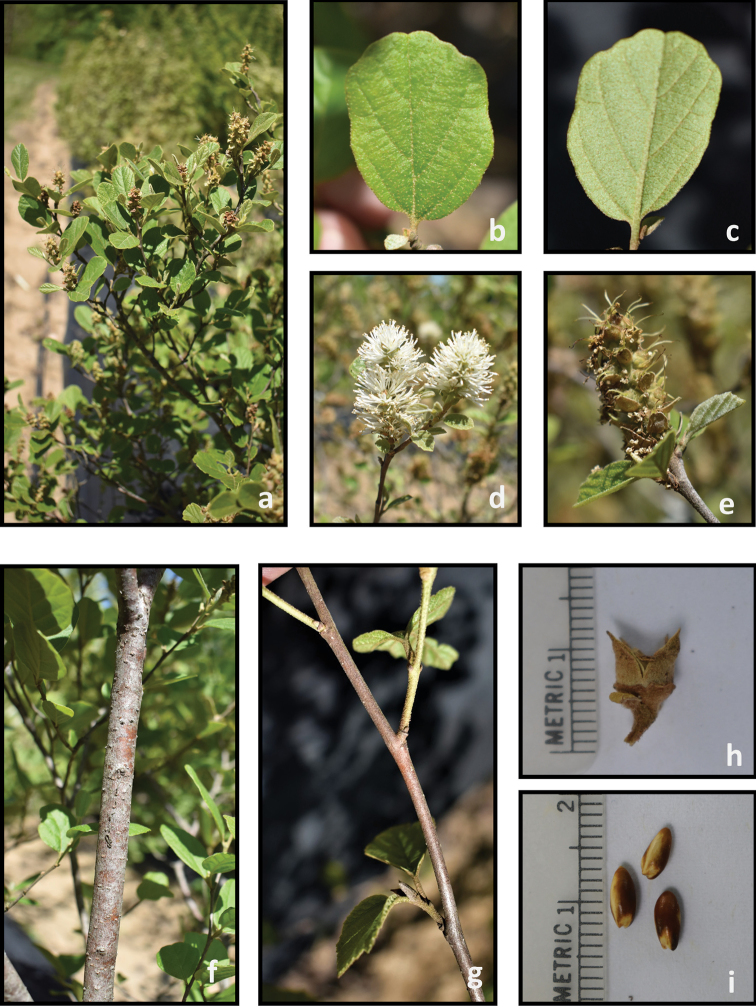
*Fothergilla gardenii***a** plant form and leaf orientation **b** adaxial leaf surface **c** abaxial leaf surface **d** inflorescence **e** young infructescence **f** stem **g** twig **h** capsule, and **i** seeds. Photos by J. Haynes of plants at the Mountain Horticultural Crops Research and Extension Center, Mills River.

Habitat in Carolina, *Garden s.n.* (***Lectotype***: LINN-693.1, LINN [online!], designated by Reveal in Jarvis et al. 1993). Figs 5, 9.

##### Description.

***Shrub***, rhizomatous, usually <1m in height; clump forming, multi-stemmed, branching. ***Leaves***: stipules ovate to lanceolate, 1.8–4.6(–7.7) × 0.9–2.7(–4.3) mm; petioles 3.9–10.5 mm long, usually ¾ the length of the IL or longer, brown-yellow pubescent; blades mostly spreading, green, narrowly ovate to ovate, (1.8–)2.8–8.7 × (1.0–)2.5–5.3 cm, pinnately 8–11-veined, bases asymmetrical or symmetrical, usually rounded to cuneate, sometimes shallowly cordate, margins crenate to serrate above middle, teeth 3–10, apices acute to obtuse, both surfaces stellate-pubescent, rarely glabrous, abaxial surface sometimes glaucous, IW:IL < or = 0.49 (*x̄* = 0.33). ***Inflorescences*** appearing before leaves, spikes terminal, appearing lateral on short lower branches, sessile or on short peduncles. ***Flowers***: stamens 10–24, filaments 3.6–13.8 mm long. ***Capsules*** 6.6–9.0 × 5.5–6.6 mm. ***Seeds*** white to mottled brown or red-brown, ellipsoid to slightly ovoid, 5.1–5.8 × 2.5–3.5 mm, apices mostly obtuse. ***Genome size and ploidy*** 3.33–3.76 pg, tetraploid (2n = 4x = 48).

##### Phenology.

Flowering beginning late Mar; fruiting by late Apr through Jul.

##### Distribution and habitat.

This species can be found along the Atlantic coastal plains of North Carolina, South Carolina, and Georgia (Fig. 5). It occurs in pocosins, savannas, and ecotones. It can be found in both sandy and peaty soils from mesic to wet conditions. It has been found in association with *Acer rubrum* L., *Amelanchier obovalis* (Michx.) Ashe, *Aronia arbutifolia* (L.) Pers., *Clethra alnifolia* L., *Cyrilla racemiflora* L., *Gaylussacia dumosa* (Andrews) Torr. & A. Gray, *G. tomentosa* (A. Gray) Pursh ex Small, *Ilex coriacea* (Pursh) Chapm., *I. glabra* (L.) A. Gray, *I. vomitoria* Aiton, *Kalmia cuneata* Michx., *K. latifolia* L., *Liquidambar styraciflua* L., *Lyonia* Nutt. spp., *Magnolia virginiana* L., *Persea palustris* (Raf.) Sarg., *Pinus serotina* Michx., *Pteridium aquilinum* (L.) Kuhn, *Quercus laevis* Walter, *Q. virginiana* Mill., *Rhododendron viscosum* (L.) Torr., *Vaccinium crassifolium* Andrews, *V. myrsinites* Lam., and *Zenobia pulverulenta* (W. Bartram ex Willd.) Pollard (fide colectoris).

##### Notes.

*Fothergilla gardenii* was apparently cultivated in England as early as 1765, grown at Kew Gardens by 1789, and English and French plant nurseries were offering seeds for sale by the early 1800s (Weaver 1971).

The species appears well adapted to periodic fires and is shade intolerant. Populations found in recently burned sites appear more abundant and robust, while populations in sites that have not been burned are generally outcompeted by other plants.

*Fothergilla gardenii* s.l. is known to attract pollinators such as honey bees and bumblebees (Dumroese and Luna 2016). The authors have also observed numerous Lepidoptera visiting the sweetly-fragranced blooms. Other members of the Hamamelidaceae, such as *Hamamelis* and *Liquidambar*, also attract those pollinators, in addition to hover flies (Syrphidae) (Dumroese and Luna 2016).

##### Conservation.

*Fothergilla gardenii* s.l. is considered Vulnerable, currently ranked by NatureServe as follows: G3G4; Alabama (S1), Florida (S1), Georgia (S2), Mississippi (SNR), North Carolina (S3S4), South Carolina (SNR) (http://explorer.natureserve.org/, accessed 11 Dec 2019). In light of the removal of the diploid taxa *F. milleri* and *F. parvifolia* from the broader historical concept of *F. gardenii*, the conservation status of this species, as well as that of the diploid taxa, warrants re-evaluation; such re-evaluation is likely to result in a more imperiled ranking than the G3G4 current assessment, following removal of a significant number of populations and a narrower distribution following its narrower taxonomic circumscription. Due to its sensitive status, we provide only skeletal collections data below.

##### Additional specimens seen

[(V) = vegetative only, (FL) = in flower, (FR) = in fruit].

Georgia. Effingham: 2012-078, 15 Apr 2014 (FL), *Lynch 43* (NCSC); 2012-078, 13 Jun 2014 (V), *Phillips 49* (NCSC^m^); 12 Jul 1997 (V), *Sorrie 9349* (NCU^m^). McDuffie: Jun 1911 (V), *Bartlett 2636* (NCU, VDB^m^).

North Carolina. Beaufort: 27 Mar 1949 (FL), *Coker s.n.* (NCU); 14 May 1966 (FL), *Blair 415* (NCSC); 21 Jun 1965 (FR), *Sawyer 2475* (NCU^m^); 5 Nov 1956 (V), *Ahles & Leisner 21471* (NCU^m^); 20 Apr 1957 (FL), *Ahles & Ramseur 23593* (NCU^m^); 31 May 2003 (FR), *Horn 4437* (BRIT^m^); 25 Jan 1937 (FL), *Melvin 3613* (BRIT^m^); 7 Jul 1994 (V), *Nifong 400* (NCU^m^). Brunswick: 8 Jun 1951 (FR), *Boyce & Wells 1656* (NCSC); 13 May 1950 (FL), *Godfrey & Wiebe 50341* (BRIT^m^, NCSC, NCU); 18 Apr 1999 (FL), *Hill 31327* (BRIT); 6 Mar 1974 (V), *Kologiski 54* (NCSC^m^); 7 May 1974 (FL), *Kologiski 125* (NCSC); 23 Jul 1974 (V), *Kologiski 311* (NCSC); 6 Jun 1975 (FR), *Kologiski 423* (NCSC); 27 Apr 1976 (FL), *Kologiski 551* (NCSC); 18 Apr 1965 (FL), *Mullen s.n.* (NCSC). Carteret: 2011-096, 26 Mar 2012 (FL), *Lynch 9* (NCSC); 2011-096, 2 Jul 2012 (V), *Lynch 76* (NCSC^m^); 2011-096, 13 Jun 2014 (FR), *Phillips 50* (NCSC); 2011-103, 21 Mar 2012 (FL), *Lynch 3* (NCSC); 2011-103, 2 Jul 2012 (V), *Lynch 75* (NCSC^m^); 22 May 1976 (V), *Wilson 1792* (NCU^m^); 9 Apr 1977 (FL), *Wilson 3067* (NCU). Columbus: 25 Apr 1958 (FL), *Bell 11417* (NCU). Craven: 19 Apr 1958 (Fl), *Radford 31924* (NCU). Cumberland: 11 Oct 1957 (V), *Ahles 36621* (NCU^m^); 28 Apr 1933 (FL), *Tallin & Harbison s.n.* (NCU). Duplin: 27 Apr 1957 (V), *Ahles & Ramseur 23990* (VDB^m^); 27 Apr 1957 (FR), *Radford & Ramseur 23990* (NCU^m^). Harnett: 10 Apr 1957 (FL), *Laing 843* (NCU); 30 Jun 2005 (FR), *Sorrie 11634* (NCU^m^). Hoke: 2011-097, 21 Mar 2012 (FL), *Lynch 4* (NCSC); 2011-097, 2 Jul 2012, (V), *Lynch 72* (NCSC^m^); 2011-097, 13 Jun 2014 (FR), *Phillips 71* (NCSC); 16 May 1976 (V), *Kral 58099* (VDB ^m^); 26 Jun 1975 (FR), *Ahles & Haesloop 29627* (NCU^m^). Lee: 19 Apr 1958 (FL), *Stewart 149* (NCU); 7 Jun 1958 (FR), *Stewart 451* (NCU^m^). Montgomery: 9 Oct 1956 (V), *Radford 19633* (NCU^m^). Moore: 17 Jul 1942 (FR), *Wicker s.n.* (NCU^m^); 8 Apr 1973 (FL), *Carter 449* (NCU); 24 Apr 1949 (FL), *Woods 2256* (NCSC). New Hanover: 6 Jun 1929 (FR), *Wells s.n.* (NCSC^m^); 29 Jun 1963 (V), *McCrary 607* (NCU ^m^); 30 Mar 1991 (FL), *Pyne & Seneca 91-013* (NCSC). Onslow: 11 May 1948 (FR), *Boyce & Moreland 647* (NCSC); 28 Apr 1951 (FL), *Beaman s.n.* (NCSC); 2 June 1948 (FR), *Boyce & Moreland 700* (NCSC^m^); 24 Jun 1965 (FR), *Wilbur 8398* (BRIT^m^). Pamlico: 12 Oct 1957 (V), *Radford 42285* (NCU^m^). Pender: 17 May 1925 (FR), *A.C.W. s.n.* (NCSC); 1 Jun 1945 (V), *Wells s.n.* (NCSC); 19 Apr 1957 (FL), *Ahles & Ramseur 23440* (NCU); 25 Apr 1947 (FL), *Fox & Wells 162* (NCSC); 12 May 1951 (FR), *Fox 4621* (NCSC); 26 Aug 1983 (V), *Leonard 8199* (UWFP^m^); 14 Apr 1925 (FL), *Wells s.n.* (NCSC). Richmond: 2011-123, 11 Apr 2012 (FL), *Lynch 13* (NCSC); 2011-123, 21 Aug 2012 (V), *Lynch 74* (NCSC^m^); 2011-085, 21 Mar 2012 (FL), *Lynch 5* (NCSC); 2011-085, 2 Jul 2012 (V), *Lynch 73* (NCSC); 2011-085, 13 Jun 2014 (V), *Phillips 55* (NCSC^m^); 19 May 2007 (FL), *Boyle 1* (NCU). Robeson: 18 Apr 1956 (FL), *Terrell 3019* (NCU). Sampson: 10 Apr 1938 (FL), *Godfrey 3394* (NCSC). Scotland: 20 Apr 1999 (FL), *Hill 31345* (BRIT^m^); 20 Jun 1957 (FR), *Ahles & Haesloop 28637* (NCU); 20 Jun 1957 (FR), *Ahles & Haesloop 28601* (NCU^m^); 4 Jun 2004 (FR), *Sorrie 11264* (NCU^m^).

South Carolina. Berkeley: 9 Apr 1944 (FR), *Duncan 5923* (NCSC). Charleston: 2012-075, 15 Apr 2014 (FL), *Lynch 42* (NCSC); 2012-075, 13 Jun 2014 (FR, V), *Phillips 48* (NCSC ^m^); 2012-77, 15 Apr 2014 (FL), *Lynch 37* (NCSC); 2012-077, 21 Aug 2012 (V), *Lynch 30* (NCSC^m^); 2012-77, 13 Jun 2014 (FR), *Phillips 53* (NCSC); 2 Apr 1944 (FL), *Duncan 5885* (NCSC). Chesterfield: 25 Apr 1968 (FR), *Ewel 666* (NCSC); 5 Jun 1956 (V), *Radford 12431* (NCU^m^); 5 Apr 1968 (FL), *Leonard & Radford 1219* (NCU). Clarendon: 20 Apr 1957 (FL), *Radford 21097* (NCU). Colleton: 5 Apr 1956 (FL), *Bell 1862* (NCU); 17 Apr 1974 (FL), *Hardin 13420* (NCSC, VDB^m^). Darlington: 25 Mar 1935 (Fl), *Matthews s.n.* (NCU); 26 Mar 1935 (FL), *Matthews & Smith s.n.* (NCU); 10 Apr 1940 (FL), *Smith 1378* (NCU). Dillon: 7 Apr 1940 (FL), *Radford & Stewart 56* (NCU). Dorchester: 20 Jul 1957 (V), *Ahles & Leisner 31966* (NCU^m^). Georgetown: 18 Apr 1987 (FL), *Taggart 78* (NCU); Near Georgetown, 28 Jul 1928 (FR), *Ashe s.n.* (NCU^m^). Horry: 2012-076, 15 Apr 2014 (FL), *Lynch 41* (NCSC); 2012-076, 13 Jun 2014 (FR, V), *Phillips 51* (NCSC ^m^). Lee: 26 Jul 1957 (V), *Radford 27396* (NCU^m^). Marlboro: 4 May 1968 (FL), *Leonard & Radford 1218* (NCU).

#### 2. Fothergilla major

Taxon classificationPlantaeSaxifragalesHamamelidaceae

Lodd., Bot. Cab. 16: Pl. 1520. 1829

8DC644F4-B01B-567E-B9EB-DF10E90AE717

F. monticola Ashe, Bot. Gaz. 24: 374. 1897. Type. North Carolina, mountains, *W.W. Ashe 1509* (***Lectotype***: MO-247915 [online!], designated here; ***isolectotypes***: DOV [online!]; OS [online!], US [online!])

##### Type.

**Figure 6.**
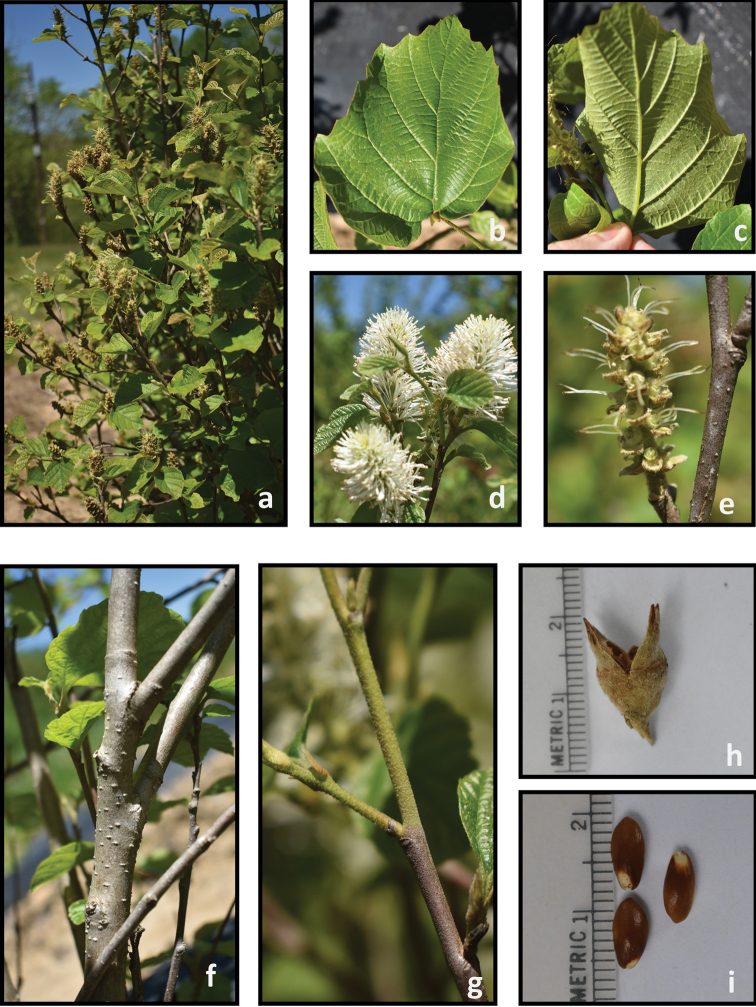
*Fothergilla major***a** plant form and leaf orientation **b** adaxial leaf surface **c** abaxial leaf surface **d** inflorescence **e** young infructescence **f** stem **g** twig **h** capsule, and **i** seeds. Photos by J. Haynes of plants at the Mountain Horticultural Crops Research and Extension Center, Mills River.

*C. Loddiges* Illustration Pl. 1520. Figs 6, 9.

##### Description.

***Shrub***, erect, robust, frequently >1 m tall (to 8 m); stems in clumps of 3 or more, branching. ***Leaves***: stipules ovate to lanceolate, 3.5–11.8 × 1.9–4.0 mm; petioles 8.3–17.1(–18.9) mm, ½ as long or longer than the IL, brown-yellow pubescent; blades mostly spreading, green, broadly ovate or elliptic to suborbiculate, rarely obovate, 3.6–13.7 × 3.2–13.0 cm, most within the upper end of those ranges, pinnately 7–12-veined, bases oblique, occasionally symmetrical, usually V-cordate, rarely nearly rounded, margins crenate or serrate to nearly entire, toothed from at or below middle to the apex, teeth 11–24, apices acute to obtuse, often pubescent, both surfaces glabrous to sparsely stellate-pubescent, abaxial surface sometimes glaucous, IW:IL > or = 0.51 (*x̄* = 0.96). ***Inflorescences*** usually appearing with leaves, spikes sessile or on short peduncles. ***Flowers***: stamens 14–32, filaments 7.3–16.2 mm long. ***Capsules*** 6.8–12.7 × 6.5–8.6 mm. ***Seeds*** nearly white to completely brown or red-brown, usually ellipsoid, 5.3–7.4 × 2.7–3.6 mm, apices rounded to obtuse. ***Genome size and ploidy*** 5.21–5.25 pg, hexaploid (2n = 6x = 72).

##### Phenology.

Flowering beginning late Mar; fruiting late May through mid-Jul.

##### Distribution and habitat.

This species can be found in the mountains and Piedmont of Alabama, Arkansas, Georgia, North Carolina, South Carolina, and Tennessee (Fig. 5). Habitat in which this species is found is variable, from rocky/xeric to peaty/mesic, but generally upland deciduous forest (typically oak-dominated) with mountain and Piedmont-associated species. However, in the Uwharrie National Forest, Montgomery County, North Carolina, *F. major* can be found growing in seepage community with Coastal Plain-affinity species. In this habitat, where burning is frequent, this species may be <1 m in height. However, height variance is apparently not simply a factor of fire frequency. *Fothergilla major* varies considerably in habit, ranging from colonial shrubs less than 2 m tall, typically found growing on sandy dampish sites in the southwestern part of the range and lower elevations (for example populations from Marshall Co., Alabama and Blount Co., Alabama) to larger tree forms growing to 6 m, with offsets but no rhizomes, typically found in more upland mountain dry forest sites with igneous geology found to the North and East (for example populations in Scott Co., Tennessee). Commonly associated upland species include *Acer rubrum*, *Aesculus sylvatica* W. Bartram, *Alnus serrulata*, *Aronia arbutifolia* (Aiton) Willd., *Calycanthus floridus* L., *Carpinus caroliniana* Walter, *Cornus florida* L., *Gaylussacia frondosa* (L.) Torr. & A. Gray, *Hamamalis virginiana* L., *Hepatica americana* (DC.) Ker Gawl, *Hypericum nudiflorum* Michx. ex Willd., *Ilex opaca* Aiton, *Kalmia latifolia*, *Liquidambar styraciflua*, *Liriodendron tulipifera* L., *Magnolia acuminata* (L.) L., *Polystichum acrostichoides* (Michx.) Schott, *Quercus alba* L., *Q. rubra* L., *Rhododendron catawbiense* Michx., and *Stewartia ovata* (Cav.) Weath. (fide colectoris).

##### Conservation.

*Fothergilla major* is considered Vulnerable rangewide, ranked by NatureServe as follows: G3; Alabama (S2), Arkansas (S1), Georgia (S1), North Carolina (S3), South Carolina (S2), Tennessee (S2); http://explorer.natureserve.org/, accessed 11 Dec 2019). Due to its sensitive status, we provide only skeletal collections data below.

##### Additional specimens seen

[(V) = vegetative only, (FL) = in flower, (FR) = in fruit].

Alabama. Blount: 2011-093, 2 Jul 2012 (V), *Lynch 91* (NCSC^m^). Cherokee: 12 Apr 1969 (FL), *Kral 34269* (NCU, VDB); 10 May 1970 (FL), *Kral 39052* (BRIT^m^); 14 Jul 1966 (FR), *Clark & Landers 5122* (BRIT^m^, NCSC). Cullman: 17 Apr 1931 (FL), *Ashe s.n.* (NCU); 16 Apr 1924 (FR), *Wolf s.n.* (AUA^m^); 4 Jul 1911 (FR), *Wolf 813* (AUA^m^); 28 May 1931 (V), *Wolf s.n.* (VDB). DeKalb: 2008-009, 21 Mar 2012 (FL), *Lynch 7* (NCSC); 2008-009, 2 Jul 2012 (V), *Lynch 81* (NCSC^m^); 14 Jul 1966 (FR), *Clark & Landers 5023* (BRIT ^m^, NCU); 22 May 1972 (FL), *Whetstone 1935* (NCU^m^). Marshall: 2011-092, s.d. (FL), *Lynch 79* (NCSC); 2011-092, 2 Jul 2012 (V), *Lynch 17* (NCSC^m^); 2011-147, 21 Aug 2012 (V), *Lynch 24* (NCSC^m^); 15 Apr 1973 (FL), *Kral 49636* (AUA, VDB ^m^). St. Clair: 31 May 1947 (FL,FR), *McVaugh 8594* (BRIT ^m^): Unspecified: n.d. (FL), *Buckley s.n.* (FLAS).

Arkansas. Searcy: 2011-082, 26 Mar 2012 (FL), *Lynch 10* (NCSC); 2011-082, 2 Jul 2012 (FR), *Lynch 80* (NCSC^m^).

Georgia. Bartow: 30 Mar 1951 (FL), *Duncan & McDowell 12200* (NCU); 30 Mar 1951 (FL), *Duncan & McDowell 12200* (BRIT, FLAS, NCSC); 6 May 1951 (FL), *Duncan & Venard 12339* (BRIT ^m^). Fulton: 2011-169, 21 Aug 2012 (V), *Lynch 28* (NCSC). Lumpkin: 2011-164, 21 Aug 2012 (V), *Lynch 27* (NCSC^m^). Walker: 2011-146, 21 Aug 2012 (V), *Lynch 25* (NCSC); 15 Apr 1986 (FL), *Coile 4547* (NCSC).

Illinois. DuPage: Cultivated at Morton Arboretum, 9 May 1990 (FL), *Gavalak 3306V90* (BRIT ^m^).

Massachusetts. Suffolk: Cultivated at Arnold Arboretum, Harvard University, 13 May 1968 (FL), *Dewolf & Bruns 2235* (BRIT).

Michigan. Ingham: Cultivated at Michigan State University, 9 May 1979 (FL), *Gillis 15041* (BRIT ^m^).

North Carolina. Burke: 2011-105, 2 Jul 2012 (V), *Lynch 81* (NCSC^m^); 7 Oct 1966 (V), *Downs 408* (NCSC); 2 Aug 1977 (FR), *Kral 60712* (VDB^m^); 25 Aug 1989 (FR), *Lance & Wood s.n.* (NCU^m^); 1 Sep 1952 (FR), *Radford 6676* (NCU^m^); 9 Sep 1976 (V), *Smith 182* (NCSC); 5 Jul 1940 (FR), *Stewart 1554* (NCU); 27 May 1964 (FR), *Wilbur 7012* (VDB^m^). Chatham: 25 Apr 1988 (FL), *Swab 75* (NCSC); 4 May 1988 (FL), *Swab 97* (NCSC); 28 May 1988 (FR), *Swab 236* (NCSC). Gaston: 19 May 1919 (FR), *Coker s.n.* (NCU^m^). Harnett: 18 Apr 2006 (V), *Sorrie & Hart 11770* (NCU^m^). Montgomery: 2011-122, 21 Aug 2012 (V), *Lynch 23* (NCSC^m^); 11 Oct 2002 (FR), *Diamond 1606* (NCU^m^); 14 Jul 2004 (V), *Schwartzman 30* (NCU^m^); 20 Jul 2004 (V), *Weakley s.n.* (NCU^m^); 15 Oct 2004 (V), *Weakley s.n.* (NCU). Orange: 2011-124, 19 Apr 2012 (FL), *Lynch 14* (NCSC); 2011-124, 2 Jul 2012, *Lynch 78* (NCSC^m^); 2011-124, 24 Jun 2014 (FR), *Phillips 62* (NCSC); Apr 1899 (FL), *Ashe s.n.* (NCU); Jun 1899 (FR), *Ashe s.n.* (NCU); 31 Apr 200 (FL), *Wally & Wichmann 125* (NCU^m^); 4 May 1910 (FR), *Clerces s.n.* (NCU). Person: 7 Jul 2005 (FR), *LeGrand s.n.* (NCU^m^). Polk: 30 May 1930 (FL), *Ashe s.n.* (NCSC); 20 Apr 1897 (FL), *Biltmore 6565* (BRIT^m^); 20 Apr 1897 (FL), *Biltmore 708* (NCU). Randolph: 22 Apr 1958 (FL), *Melvin s.n.* (NCU). Rutherford: 2011-163, 21 Aug 2012 (V), *Lynch 26* (NCSC^m^); 2011-163, 15 Apr 2013 (FL), *Lynch 39* (NCSC); 2011-163, 24 Jun 2014 (FR), *Phillips 64* (NCSC); 21 Apr 1956 (FL), *Bell 2118* (NCU). Stokes: 23 Apr 1950 (FL), *Fox et al. 3565* (NCSC); s.d. (FR), *Harbison s.n.* (NCU); 5 May 1936 (FL), *Hunt 13474* (BRIT ^m^, NCU); 7 Jul 1969 (FR), *Leonard & Russ 2553* (NCU^m^); 21 Apr 1974 (FL), *Massey 3900* (VDB); 26 Apr 1958 (FL), *Matthews 54* (BRIT); 15 Jun 1967 (FR), *Radford 45392* (AUA^m^, MISS, NCSC, NCU); 4 Jun 1958 (FR), *Radford 34675* (VDB ^m^); 9 Apr 1933 (FL), *Schallert 3613* (BRIT). Surry: 19 May 1935 (V), *Harbison s.n.* (NCU). Transylvania: 2011-112, 2 Jul 2012 (V), *Lynch 16* (NCSC); 2011-112, 24 Apr 2014 (FL), *Lynch 47* (NCSC); 2011-112, 24 Jun 2014 (FR), *Phillips 63* (NCSC); 2011-131, 21 Aug 2012 (FR), *Lynch 22* (NCSC^m^); 19 Jun 1965 (FR), *Eggers 1262* (VDB ^m^); 26 Jul 1962 (FR), *Rodgers 62064a* (NCU^m^); 24 May 2006 (FR), *Schwartzman 29* (NCU^m^). Wake: 28 Oct 2005 (V), *Bruhn s.n.* (NCU).

Pennsylvania. Marion: Cultivated at the Arboretum of the Barnes Foundation, 27 Apr 1968 (FL), *Fogg 1* (UWFP).

South Carolina. Greenville: 17 May 1976 (FR), *Kral 58130* (VDB ^m^); 15 Oct 1988 (V), *Hill 20066* (BRIT ^m^); 23 May 1996 (V), *Townsend 874* (VDB ^m^). Oconee: 2011-091, 28 Mar 2012 (FL), *Lynch 12* (NCSC); 2011-091, 2 Jul 2012 (V), *Lynch 77* (NCSC^m^); 26 Apr 1965 (FL), *Radford 44707* (NCU); 20 Apr 1973 (FL), *Rogers & Green 73078* (FLAS-2). Pickens: 15 June 1992 (FT), *Hill 23387* (BRIT ^m^);

Tennessee. Grainger: 20 Apr 1931 (FL), *Sharp 556* (BRIT, NCSC); 30 Apr 1931 (FL), *Jennison & Sharp s.n.* (NCU); May 3 1936 (Fl), *Sharp & Underwood 4205* (NCU^m^, NCSC). Greene: 17 May 1970 (FL), *Sharp et al. 45204* (BRIT ^m^, NCU, VDB). Scott: 2012-065, 21 Aug 2012 (V), *Lynch 29* (NCSC ^m^); 2012-065, 15 Apr 2014 (FL), *Lynch 44* (NCSC); 23 Apr 1972 (FL), *Clebsch s.n.* (VDB); 19 Apr 1979 (FL), *Whitten & Noss s.n.* (FLAS-2); 23 Jun 1978 (FR), *Wofford et al. 78-112* (VDB ^m^). Sevier: 1 May 1960 (Fl), *Sharp 26818* (BRIT, NCU); 25 Apr 1970 (FL), *Williams 82* (AUA ^m^); 25 Apr 1970 (FL), *Pippin 83* (VDB); 2 Apr 1938 (FL), *Jennison 53* (VDB ^m^); 26 Apr 1957 (FL), *Sharp 21575* (BRIT ^m^).

Washington D.C. U.S. National Arboretum, 16 Oct 1990 (V), *Meyer 37144* (FLAS).

#### 3. Fothergilla milleri

Taxon classificationPlantaeSaxifragalesHamamelidaceae

W.D.Phillips & J.E.Haynes sp. nov.

DC5E3CB6-8D0A-57AA-96CC-7D6838D9DC9F

urn:lsid:ipni.org:names:77208304-1

##### Type.

**Figure 7.**
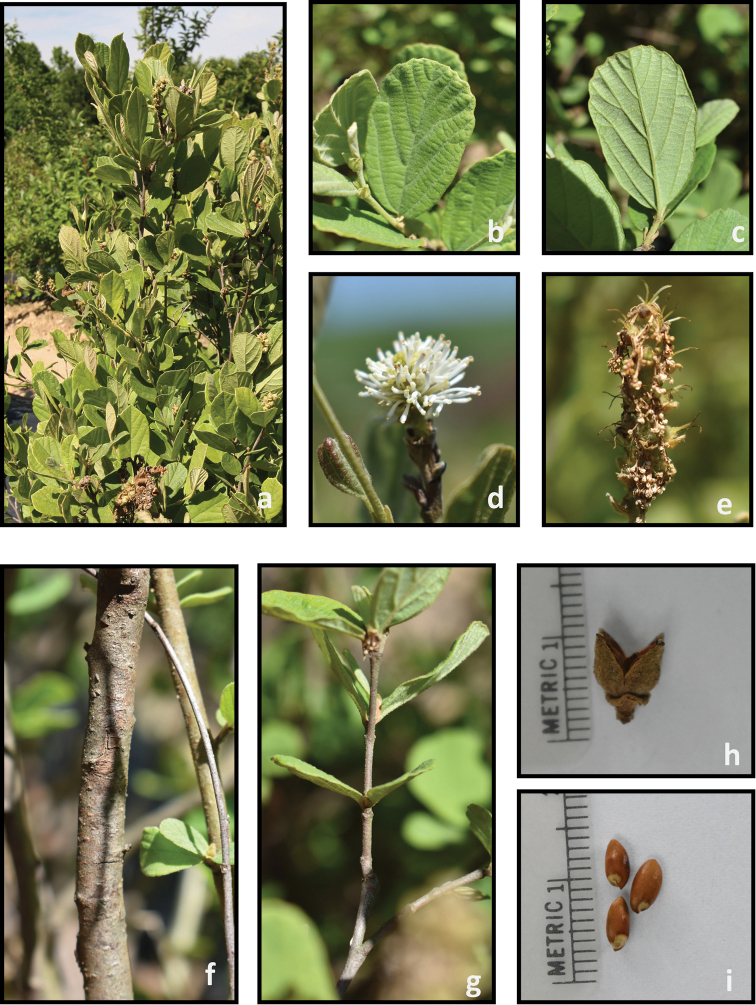
*Fothergilla milleri***a** plant form and leaf orientation **b** adaxial leaf surface **c** abaxial leaf surface **d** inflorescence **e** young infructescence **f** stem **g** twig **h** capsule, and **i** seeds. Photos by J. Haynes of plants at the Mountain Horticultural Crops Research and Extension Center, Mills River.

Florida. Walton Co.: Voucher specimen from containerized plant at Mountain Horticultural Crops Research and Extension Center, Mills River, NC (leaves and stem collected 19/7/2012), [living plant originally collected 25 Mar 2011 by Ron Miller from Walton Co., Florida], 2011-088, 19 Jul 2012 (V), *Nathan Lynch 70* (***Holotype***: NCSC-00102544^m^, here designated). Figs 1C, 3A, 7, 9.

##### Diagnosis.

*Fothergilla milleri* is morphologically most similar to *F. gardenii*, but differs from the latter by leaves held erect (vs. spreading in *F. gardenii*), blades blue-green or gray-green (vs. green in *F. gardenii*), petioles 1/3–1/2 the length of the IL (vs. ¾ the length of the IL or longer in *F. gardenii*), and seed apices acute to acuminate (vs. rounded or obtuse in *F. gardenii*).

##### Description.

***Shrub***, rhizomatous, erect, to 1.5 m tall, clump-forming, multi-stemmed, branching. ***Leaves***: stipules ovate to lanceolate, 2.5–7.8 × 1.0–3.0 mm; petioles 2.6–8.0 mm long, usually 1/3–1/2 the length of the IL, brown-yellow pubescent; blades erect, blue-green to gray-green, obovate, 3.2–8.0 × 3.0–4.8 cm, pinnately 8–10-veined, bases rounded to truncate, margins crenate to serrate above middle and mostly near the apex, sometimes appearing crenate to entire, apices mostly obtuse to acute, both surfaces conspicuously stellate-pubescent, sometimes sparsely so, abaxial surface not glaucous, IW:IL < or = 0.48 (*x̄* = 0.29). ***Inflorescences*** appearing before leaves, spikes on short peduncles or sessile. ***Flowers***: stamens 15–22, filaments 4.3–10 mm long. ***Capsules*** 7.0–9.0 × 5.0 –7.0 cm. ***Seeds*** usually completely brown to red-brown, ovoid, 4.5–6.2 × 2.4–3.2 mm, apices acuminate, often recurved. ***Genome size and ploidy***: 1.70–1.78 pg, diploid (2n = 2x = 24).

##### Phenology.

Flowering beginning late Mar; fruiting late May through the end of Jul.

##### Distribution and habitat.

This species can be found in the panhandle of Florida, in Alabama in the Gulf coastal plain, and is also known from one county in Georgia (Fig. 5). This species is found in sandy peat shrub bogs, seepages, dry longleaf pine woodlands, and the edge of *Cyrilla racemiflora* or *Taxodium ascendens* swamp forests. Plant associates include *Acer rubrum*, *Arundinaria tecta* (Walter) Muhl., *Clethra alnifolia*, *Hibiscus aculeatus* Walter, *Hypericum cistifolium* Lam., *Juncus trigonocarpus* Steud., *Dichanthelium scoparium* (Lam.) Gould., *Osmundastrum cinnamomeum* (L.) C. Presl, *Rhexia virginica* L., *Rhynchospora chalarocephala* Fernald & Gale, and *Xyris fimbriata* Elliott (fide colectoris).

##### Etymology.

The species is named in honor of Dr Ron Miller, Pensacola, Florida, who championed this project, originally suggested that diploid cytotypes might still exist, and ultimately found them. Dr Miller’s extensive effort and field work (with colleagues) provided the foundation for this research and establishment of ex situ living collections, including accessions in the U.S. National Plant Germplasm System.

##### Conservation.

The conservation status of this species must be reassessed. It is presently known from only seven counties, and would appear to have an imperilment status at least as severe as that of *F. major*. Consequently, only skeletal collections data are provided below.

##### Additional specimens seen

[(V) = vegetative only, (FL) = in flower, (FR) = in fruit].

Alabama. Baldwin: 2011-087, 21 Mar 2012 (FL), *Lynch 1* (NCSC); 2011-087, 2 Jul 2012 (V), *Lynch 68* (NCSC^m^); 2011-087, 13 Jun 2014 (FR), *Phillips 58* (NCSC); 16 Jun 1984 (FR), *Hedges 156* (UWFP^m^); 19 Apr 2001 (FR), *Schotz 1830* (UNA [online!]). Covington: 25 Jun 1906 (FR), *Harper 108* (NY [online!]). Escambia: 11 Apr 1964 (FL), *Kral 19698* (AUA^m^). Geneva: 25 Jul 1968 (V), *Kral 32029* (VDB^m^); 26 Apr 1969 (FL), *Kral 34524* (VDB^m^); 7 Sep 1994 (V), *Simmers s.n.* (HTTU [online!]). Monroe: 15 Jun 1959 (V), *Grelen s.n.* (FLAS^m^).

Florida. Okaloosa: 2011-083, 21 Mar 2012 (FL), *Lynch 2* (NCSC); 2011-083, 2 Jul 2012 (FL), *Lynch 67* (NCSC); 2011-083, 13 Jun 2014 (FR), *Phillips 57* (NCSC^m^); 20 Apr 1991 (FL), *Burkhalter 12638* (FLAS^m^, UWFP); 5 Aug 1990 (V), *Burkhalter 12211* (UWFP^m^); 13 Jul 1996 (V), *Burkhalter 15064* (UWFP); 18 May 1993 (FR), *Naczi 3028* (KNK [online!]). Walton: 8 Apr 1899 (FL), *Biltmore Herbarium 7609b* (FLAS^m^); 2011-088, 28 Mar 2012 (FL), *Lynch 11* (NCSU); 2012-060, 19 Mar 2013 (FL), *Lynch 34* (NCSC); 2012-060, 2 Jul 2012 (V), *Lynch 15* (NCSC); 2012-060, 13 Jun 2014 (FR) *Phillips 59* (NCSC); 18 Aug 1990 (V), *Orzell and Bridges 14757* (FLAS^m^, NY [online!], USF [online!]).

Georgia. Taylor: 2011-178, 26 Mar 2012 (FL), *Lynch 8* (NCSC); 2011-178, 2 Jul 2012 (V), *Lynch 69* (NCSC^m^).

#### 4. Fothergilla parvifolia

Taxon classificationPlantaeSaxifragalesHamamelidaceae

Kearney in Small, Fl. S.E. U.S. 509, 1331. 1903

988E10E7-FD07-532B-8B40-80EC41C51904

##### Type.

**Figure 8.**
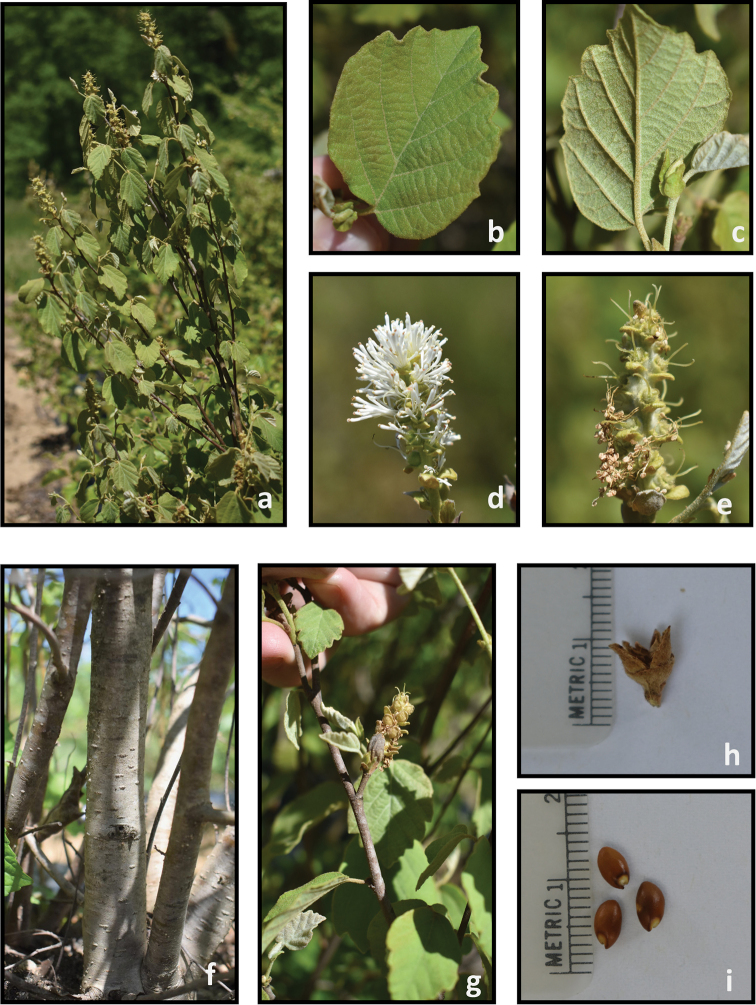
*Fothergilla parvifolia***a** plant form and leaf orientation **b** adaxial leaf surface **c** abaxial leaf surface **d** inflorescence **e** young infructescence **f** stem **g** twig **h** capsule, and **i** seeds. Photos by J. Haynes of plants at the Mountain Horticultural Crops Research and Extension Center, Mills River.

**Figure 9.**
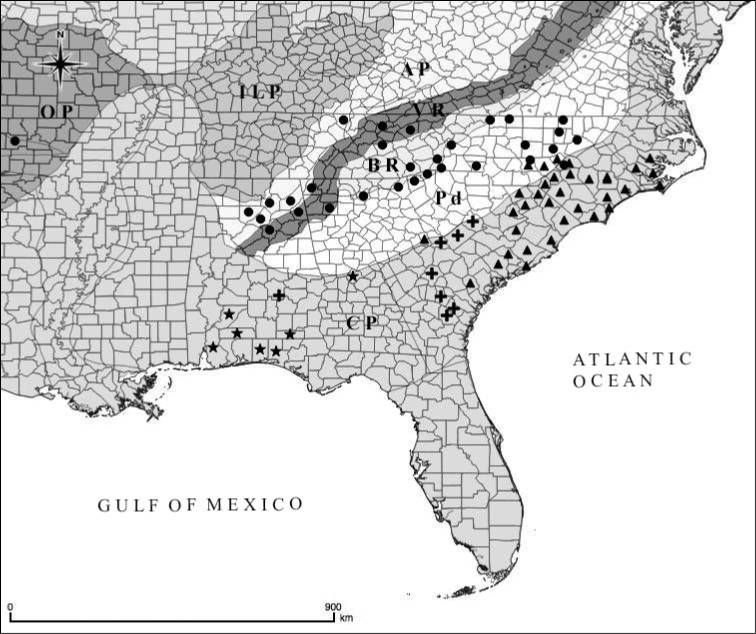
Distribution of *Fothergilla* in the southeastern United States. Circle = *F. major* (6x); triangle = *F. gardenii* (4x); star = *F. milleri* (2x); plus sign = *F. parvifolia* (2x). Physiographic provinces follow Fenneman and Johnson (1946), shape files courtesy of usgs.gov. AP = Appalachian Plateau; BR = Blue Ridge; CP = Coastal Plain; ILP = Interior Low Plateau; OP = Ozark Plateau; Pd = Piedmont; VR = Valley and Ridge. Map generated by A. Krings in QGIS (QGIS Development Team 2019).

Georgia. Wayne Co., Jesup, dry soil, 4 June 1893 (FR), *Kearney s.n.* (***Holotype***: NY!; ***isotype***: F [online!]). Figs 1B, 3B, 8, 9.

##### Description.

***Shrub***, rhizomatous, erect, to 1 m tall, clump-forming, multi-stemmed, branching. ***Leaves***: stipules ovate to lanceolate, 3.5–6.0 × 8.0–13.0 mm, curved downward; petioles 4.4–17.8 mm long, usually nearly as long to longer than the IL, brown-yellow pubescent; blades drooping, green, mostly ovate, 3.2–12.1 × 3.0–6.2 cm, pinnately 8–10-veined, bases cordate, margins crenate to serrate from middle, apices acute, adaxial and abaxial surfaces not glaucous, stellate-pubescent, sometimes sparsely so, IW:IL > or = 0.62 (*x̄* = 0.96). ***Inflorescences*** appearing before leaves, spikes on short peduncles or sessile. ***Flowers***: stamens 15–20 per flower, filaments 6.6–9.3 mm long. ***Capsules*** 7.5–10.0 × 5.0 –7.6 mm. ***Seeds*** usually completely brown to red-brown, ovoid, apex obtuse, 4.5–6.2 × 2.4–3.2 mm, apices obtuse to acute. ***Genome size and ploidy*** 1.73–1.82 pg, diploid (2n = 2x = 24).

##### Phenology.

Flowering Mar–May; fruiting May–Sep.

##### Distribution and habitat.

This species range is restricted to Georgia, South Carolina, and Alabama (Fig. 5). Because there are few herbarium records for this species, little is known about its exact distribution and environmental restrictions. According to the few notes available on herbarium specimens, it occurs in seepages and margins of bogs, bay swamps, and watercourses.

##### Conservation.

The conservation status of this species needs to be assessed. It is presently known from only eight counties, and would appear to have an imperilment status at least as severe as that of *F. major*. Consequently, only skeletal collections data are provided below.

##### Additional specimens seen

[(V) = vegetative only, (FL) = in flower, (FR) = in fruit].

Alabama. Montgomery: 12 Sep 1899 (FR), *Harbison 1033* (NCU^m^).

Georgia. Augusta-Richmond: 2 Apr 1898 (FL), *Cuthbert s.n.* (FLAS). Brantley: 23 Aug 1947 (V), *Thorne & Norris 6284* (GEO [online!]). Emanuel: 2011-170, 2 Jul 2012 (V), *Lynch 19* (NCSC^m^); 2011-170, 15 Mar 2013 (FL), *Lynch 33* (NCSC); 2011-170, 14 Jun 2014 (FR), *Phillips 60* (NCSC). Long: 2011-171, 2 Jul 2012 (V), *Lynch 20* (NCSC^m^); 2011-171, 14 Mar 2013 (FL), *Lynch 32* (NCSC); 2011-171, 13 Jun 2014 (FR), *Phillips 61* (NCSC); Tattnall: 2011-168, 19 Mar 2013 (FL), *Lynch 35* (NCSC); 2011-168, 2 Jul 2012 (V), *Lynch 18* (NCSC^m^). Wayne: 31 Aug 1904 (V), *Biltmore Herbarium 14940* (NY [online!]).

South Carolina. Aiken: 2012-084, 15 Apr 2014 (FL), *Lynch 40* (NCSC); 2012-084, 13 Jun 2014 (V), *Phillips 56* (NCSC^m^). Lexington: 13 Sept 1988 (V), *Pittman 9139817* (BRIT^m^); 27 May 1957 (FR), *Radford 23378* (NCU^m^).

## Supplementary Material

XML Treatment for Fothergilla

XML Treatment for Fothergilla gardenii

XML Treatment for Fothergilla major

XML Treatment for Fothergilla milleri

XML Treatment for Fothergilla parvifolia
