# Corrigendum: Revision of *Fothergilla* (Hamamelidaceae), including resurrection of *F. parvifolia* and a new species, *F. milleri*

**DOI:** 10.3897/phytokeys.146.53037

**Published:** 2020-05-08

**Authors:** Jake E. Haynes, Whitney D. Phillips, Alexander Krings, Nathan P. Lynch, Thomas G. Ranney

**Affiliations:** 1 Department of Plant and Microbial Biology, North Carolina State University, Raleigh, NC 27695, USA; 2 Department of Horticultural Science, North Carolina State University, Raleigh, NC 27695, USA; 3 Mountain Horticultural Crops Research and Extension Center, Mills River, NC 28759, USA

## Abstract

Not applicable

In our recent work (Haynes et al. 2020), we overlooked a pertinent paper (Turner 2016), which affects the nomenclature and typification of *Fothergilla major*. We provide a correction below:

***Fothergilla major* (Sims) Sweet, Hort. Suburb. Lond.: 124. 1818.**

Fothergilla alnifolia var. major Sims in Bot. Mag. 33: sub t. 1341, t. 1342. 1810

**Type.** Illustration in Bot. Mag. 33: t. 1342. 1810 (Lectotype designated by Turner 2016)
